# A polybromodiphenyl ether from an Indonesian marine sponge *Lamellodysideaherbacea* and its chemical derivatives inhibit protein tyrosine phosphatase 1B, an important target for diabetes treatment

**DOI:** 10.1007/s11418-012-0735-y

**Published:** 2012-12-29

**Authors:** Hiroyuki Yamazaki, Deiske A. Sumilat, Syu-ichi Kanno, Kazuyo Ukai, Henki Rotinsulu, Defny S. Wewengkang, Masaaki Ishikawa, Remy E. P. Mangindaan, Michio Namikoshi

**Affiliations:** 1 Faculty of Pharmaceutical Sciences, Tohoku Pharmaceutical University, Sendai, 981-8558 Japan; 2 Faculty of Fisheries and Marine Science, Sam Ratulangi University, Kampus Bahu, Manado, 95115 Indonesia; 3 Faculty of Agriculture, Universitas Pembangunan Indonesia, Manado, 95361 Indonesia

**Keywords:** Protein tyrosine phosphatase 1B (PTP1B), Inhibitor, Type-2 diabetes mellitus (T2DM), Polybrominated diphenyl ether, Indonesian marine sponge, *Lamellodysideaherbacea*

## Abstract

The ethanol extract of an Indonesian marine sponge *Lamellodysidea* *herbacea* inhibited the activity of protein tyrosine phosphatase 1B (PTP1B), an important target enzyme for the treatment of type II diabetes. Bioassay-guided isolation yielded a known polybromodiphenyl ether (**1**) as a sole bioactive component. The structure of **1** was confirmed by spectroscopic data for **1** and its methyl ether derivative (**2**). Compound **1** markedly inhibited the PTP1B activity (IC_50_ = 0.85 μM) and showed a moderate cytotoxicity against two human cancer cell lines, HCT-15 (colon) and Jurkat (T-cell lymphoma) cells. On the other hand, compound **2** maintained potent inhibitory activity against PTP1B (IC_50_ = 1.7 μM) but did not show apparent cytotoxicity at 18 μM against these cancer cells. Four ester derivatives [acetyl (**3**), butyryl (**4**), hexanoyl (**5**), and benzoyl (**6**)] were prepared from **1** and their activities evaluated against PTP1B and two cancer cell lines to investigate the structure–activity relationships. Although compounds **3**–**6** exhibited potent inhibitory effects against PTP1B activity, cytotoxicity against HCT-15 and Jurkat cells was observed as a similar efficacy to that of **1**. From these results, compound **2** was found to be the best inhibitor of PTP1B with no apparent cytotoxicity. Therefore, **2** may be a lead compound for making a new type of PTP1B inhibitor. Moreover, compound **2** did not inhibit the cell growth of Huh-7 cells (hepatoma). Hepatocytes are one of the locations of PTP1B, and Huh-7 cells are used to study the mechanism of action of compound **2**.

## Introduction

Type-2 diabetes mellitus (T2DM) is a chronic metabolic disorder characterized by β-cell dysfunction and insulin resistance, and has emerged as a major health care burden around the world [1–3]. Protein tyrosine phosphatase 1B (PTP1B) is an enzyme found in the important insulin-targeted tissues such as liver, muscle, and fat cells. PTP1B plays a key role as a negative regulator in insulin signal transduction [4] by dephosphorylating activated insulin receptors (IR) or insulin receptor substrates (IRS) [5, 6]. An excess of PTP1B will impair insulin down-regulation [7–9], leading to type II diabetes mellitus. Based on the above research results, the inhibition of PTP1B has been sought as a novel therapeutic strategy, and much attention has been paid to PTP1B inhibitors using small molecules for the treatment of type II diabetes [10–12].

In our screening program to search for PTP1B inhibitors, we have tested the ethanol extracts of Indonesian marine organisms such as marine sponges and ascidians, and the extract of a marine sponge *Lamellodysidea* *herbacea* exhibited significant inhibitory activity against PTP1B. Bioassay-guided separation of the extract led to the isolation of a bioactive component, and the structure was assigned as 2-(3′,5′-dibromo-2′-methoxyphenoxy)-3,5-dibromophenol (**1**) [13]. We described herein the PTP1B inhibitory activity and cytotoxicity against two human cancer cell lines, HCT-15 (colon) and Jurkat (T-cell lymphoma), of compound **1** and its methyl ether (**2**) and ester derivatives (**3**–**6**).

## Materials and methods

### General experimental procedure

EI–MS was performed by a JMS-MS 700 mass spectrometer (JEOL, Tokyo, Japan). ^1^H- and ^13^C-NMR spectra were recorded on a JNM-AL-400 NMR spectrometer (JEOL) at 400 MHz for ^1^H and 100 MHz for ^13^C in CDCl_3_ (δ_H_ 7.26, δ_C_ 77.0). Preparative HPLC was carried out using the L-6200 system (Hitachi Ltd., Tokyo, Japan).

### Materials

Fetal bovine serum (FBS) and other culture materials were purchased from Invitrogen (Carlsbad, CA, USA). 3-(4,5-Dimethylthiazol-2-yl)-2,5-diphenyltetrazolium bromide (MTT) was purchased from Sigma-Aldrich (St. Louis, MO, USA). All other chemicals including organic solvent were purchased from Wako Pure Chemical Industries Ltd. (Osaka, Japan).

### Marine sponge

The marine sponge was collected by scuba diving in the coral reef at Manado, Indonesia, in 2010 and identified as *Lamellodysidea* *herbacea*. The voucher specimen is deposited at the Faculty of Fisheries and Marine Science, Sam Ratulangi University and the Faculty of Pharmaceutical Sciences, Tohoku Pharmaceutical University as 10-09-16=2-6.

### Extraction and isolation

The marine sponge (94 g wet weight) was thawed, cut into small pieces, and extracted three times with ethanol. The ethanol extract was evaporated to dryness (284.3 mg) and 20 mg of the crude extract was subjected to HPLC separation (90 % MeOH; detection, UV 210 nm; flow rate, 2.0 mL/min) using an ODS column (PEGASIL ODS, 10 mm × 250 mm, Senshu Scientific Co., Tokyo, Japan) to give 5.4 mg of 2-(3′,5′-dibromo-2′-methoxyphenoxy)-3,5-dibromophenol (**1**).

#### 2-(3′,5′-Dibromo-2′-methoxyphenoxy)-3,5-dibromophenol (**1**)

Obtained as a viscous oil; ^1^H-NMR (CDCl_3_) δ 4.03 (s, 3H), 6.80 (d, 1H, *J* = 4.0), 7.18 (d, 1H, *J* = 4.0), 7.35 (d, 1H, *J* = 4.0), 7.45 (d, 1H, *J* = 4.0); ^13^C-NMR (CDCl_3_) δ 61.5, 117.3, 117.3, 118.7, 119.0, 119.9, 120.1, 127.4, 130.5, 139.0, 145.9, 150.5, 150.7; EI–MS *m*/*z* 528, 530 532, 534, and 526 [M^+^]; HREI–MS *m*/*z* 527.7180 (Δ −2.7 mmu, calcd for C_13_H_8_^79^Br_4_O_3_: 527.7207), 529.7203 (Δ +1.7 mmu, calcd for C_13_H_8_^79^Br_3_^81^Br_1_O_3_: 529.7186), 531.7159 (Δ −0.7 mmu, calcd for C_13_H_8_^79^Br_2_^81^Br_2_O_3_: 531.7166), 533.7137 (Δ −0.9 mmu, calcd for C_13_H_8_^79^Br_1_^81^Br_3_O_3_: 533.7146), 535.7103 (Δ −2.2 mmu, calcd for C_13_H_8_^81^Br_4_O_3_: 535.7125).

### Preparation of methyl derivative (**2**)

TMS-diazomethane (73 μL, 0.064 mmol) was added to a MeOH solution of **1** (3.8 mg, 0.0071 mmol in 300 μL) and stirred at room temperature for 14 h. The reaction mixture was concentrated in vacuo to give a brown material, and a product was purified by preparative HPLC (90 % MeOH) using ODS column (PEGASIL ODS) to give 3,5-dibromo-2-(3′,5′-dibromo-2′-methoxyphenoxy)-1-methoxybenzene (**2**, 2.0 mg, 0.0037 mmol, 52 %).

#### 3,5-Dibromo-2-(3′,5′-dibromo-2′-methoxyphenoxy)-1-methoxybenzene (**2**)

Obtained as a viscous oil; ^1^H-NMR (CDCl_3_) δ 3.76 (s, 3H), 4.00 (s, 3H), 6.46 (d, 1H, *J* = 4.0), 7.09 (d, 1H, *J* = 4.0), 7.36 (d, 1H, *J* = 4.0), 7.42 (d, 1H, *J* = 4.0); ^13^C-NMR (CDCl_3_) δ 57.2, 61.7, 116.4, 117.2, 119.3, 119.4, 119.6, 128.2, 129.5, 137.2, 140.1, 146.2, 152.1, 154.2; EI–MS *m*/*z* 542, 544 546, 548, and 550 [M^+^]; HREI–MS *m*/*z* 541.7386 (Δ +2.2 mmu, calcd for C_14_H_10_^79^Br_4_O_3_: 541.7364), 543.7319 (Δ −2.4 mmu, calcd for C_14_H_10_^79^Br_3_^81^Br_1_O_3_: 543.7343), 545.7318 (Δ −0.5 mmu, calcd for C_14_H_10_^79^Br_2_^81^Br_2_O_3_: 545.7323), 547.7288 (Δ −1.4 mmu, calcd for C_14_H_10_^79^Br_1_^81^Br_3_O_3_: 547.7302), 549.7262 (Δ −2.0 mmu, calcd for C_14_H_10_^81^Br_4_O_3_: 549.7282).

### Preparation of derivatives **3**–**6**

Acetic anhydride (100 μL, 1.1 mmol) and 4-(dimethylamino)pyridine (1.0 mg, 0.0080 mmol) were added to a solution of **1** (3.0 mg, 0.056 mmol) in pyridine (100 μL), and the resulting solution was stirred at room temperature for 12 h. The reaction mixture was concentrated in vacuo to dryness, and a product was purified by preparative HPLC (column; PEGASIL ODS, 10 mm × 250 mm; solvent, 90 % MeOH; detection, UV at 220 nm; flow rate, 2.0 mL/min) to give 3,5-dibromo-2-(3′,5′-dibromo-2′-methoxyphenoxy)phenyl ethanoate (**3**, 1.2 mg, 0.0022 mmol, 30 %). The other derivatives (**4**–**6**) were prepared using the following regents instead of acetic anhydride: *n*-butyric anhydride (**4**, 1.4 mg, 0.0023 mmol, 32 %), *n*-hexanoic anhydride (**5**, 1.1 mg, 0.0018 mmol, 25 %), and benzoyl chloride (**6**, 1.5 mg, 0.0023 mmol, 33 %).

#### 3,5-Dibromo-2-(3′,5′-dibromo-2′-methoxyphenoxy)phenyl ethanoate (**3**)

Obtained as a viscous oil; ^1^H-NMR (CDCl_3_) δ 2.08 (s, 3H), 3.95 (s, 3H), 6.78 (d, 1H, *J* = 2.4), 7.18 (d, 1H, *J* = 2.4), 7.34 (d, 1H, *J* = 2.4), 7.44 (d, 1H, *J* = 2.4); EI–MS *m*/*z* 570, 572 574, 576, and 578 [M^+^]; HREI–MS *m*/*z* 569.7316 (Δ +0.3 mmu, calcd for C_15_H_10_^79^Br_4_O_4_: 569.7313), 571.7296 (Δ +0.4 mmu, calcd for C_15_H_10_^79^Br_3_^81^Br_1_O_4_: 571.7292), 573.7283 (Δ +1.1 mmu, calcd for C_15_H_10_^79^Br_2_^81^Br_2_O_4_: 573.7272), 575.7247 (Δ −0.4 mmu, calcd for C_15_H_10_^79^Br_1_^81^Br_3_O_4_: 575.7251), 577.7216 (Δ −1.5 mmu, calcd for C_15_H_10_^81^Br_4_O_4_: 577.7231).

#### 3,5-Dibromo-2-(3′,5′-dibromo-2′-methoxyphenoxy)phenyl butanoate (**4**)

Obtained as a viscous oil; ^1^H-NMR (CDCl_3_) δ 0.89 (t, 3H, *J* = 7.2), 1.57 (m, 2H), 2.29 (t, 2H, *J* = 7.2), 3.95 (s, 3H), 6.58 (d, 1H, *J* = 1.9), 7.36 (d, 1H, *J* = 2.4), 7.40 (d, 1H, *J* = 1.9), 7.71 (d, 1H, *J* = 2.4); EI–MS *m*/*z* 598, 600 602, 604, and 606 [M^+^]; HREI–MS *m*/*z* 597.7625 (Δ −0.1 mmu, calcd for C_17_H_14_^79^Br_4_O_4_: 597.7626), 599.7580 (Δ −2.5 mmu, calcd for C_17_H_14_^79^Br_3_^81^Br_1_O_4_: 599.7605), 601.7569 (Δ −1.6 mmu, calcd for C_17_H_14_^79^Br_2_^81^Br_2_O_4_: 601.7585), 603.7591 (Δ +2.7 mmu, calcd for C_17_H_14_^79^Br_1_^81^Br_3_O_4_: 603.7564), 605.7518 (Δ −2.6 mmu, calcd for C_17_H_14_^81^Br_4_O_4_: 605.7544).

#### 3,5-Dibromo-2-(3′,5′-dibromo-2′-methoxyphenoxy)phenyl hexanoate (**5**)

Obtained as a viscous oil; ^1^H-NMR (CDCl_3_) δ 0.87 (t, 3H, *J* = 6.8), 1.25 (m, 4H), 1.51 (m, 2H), 2.30 (t, 2H, *J* = 7.8), 3.95 (s, 3H), 6.57 (d, 1H, *J* = 2.0), 7.36 (d, 1H, *J* = 2.0), 7.40 (d, 1H, *J* = 2.0), 7.71 (d, 1H, *J* = 2.4); EI–MS *m*/*z* 626, 628 630, 632, and 634 [M^+^]; HREI–MS *m*/*z* 625.7952 (Δ +1.3 mmu, calcd for C_19_H_18_^79^Br_4_O_4_: 625.7939), 627.7924 (Δ +0.6 mmu, calcd for C_19_H_18_^79^Br_3_^81^Br_1_O_4_: 627.7918), 629.7881 (Δ −1.7 mmu, calcd for C_19_H_18_^79^Br_2_^81^Br_2_O_4_: 629.7898), 631.7874 (Δ −0.4 mmu, calcd for C_19_H_18_^79^Br_1_^81^Br_3_O_4_: 631.7878), 633.7856 (calcd for C_19_H_18_^81^Br_4_O_4_: 633.7856).

#### 3,5-Dibromo-2-(3′,5′-dibromo-2′-methoxyphenoxy)phenyl benzoate (**6**)

Obtained as a viscous oil; ^1^H-NMR (CDCl_3_) δ 3.80 (s, 3H), 6.68 (d, 1H, *J* = 2.4), 7.27 (d, 1H, *J* = 1.9), 7.41 (t, 2H, *J* = 7.7), 7.52 (d, 1H, *J* = 1.9), 7.59 (t, 1H, *J* = 7.3), 7.76 (d, 1H, *J* = 2.4), 7.83 (d, 2H, *J* = 7.2); EI–MS *m*/*z* 632, 634, 636, 638, and 640 [M^+^]; HREI–MS *m*/*z* 631.7455 (Δ −1.4 mmu, calcd for C_20_H_12_^79^Br_4_O_4_: 631.7469), 633.7468 (Δ +1.9 mmu, calcd for C_20_H_12_^79^Br_3_^81^Br_1_O_4_: 633.7449), 635.7433 (Δ +0.5 mmu, calcd for C_20_H_12_^79^Br_2_^81^Br_2_O_4_: 635.7428), 637.7430 (Δ +2.3 mmu, calcd for C_20_H_12_^79^Br_1_^81^Br_3_O_4_: 637.7407), 639.7379 (Δ −0.9 mmu, calcd for C_20_H_12_^81^Br_4_O_4_: 639.7388).

### PTP1B inhibitory assay

Protein tyrosine phosphatase 1B (PTP1B) inhibitory activity was determined by measuring the rate of hydrolysis of a substrate, *p*-nitrophenyl phosphate (pNPP, Sigma, St. Louis, MO, USA) according to the published method with a slight modification [14]. Briefly, PTP1B (100 μL of 0.5 μg/mL stock solution, Enzo Life Sciences, Farmingdale, NY, USA) in 50 mM citrate buffer (pH 6.0) containing 0.1 M NaCl, 1 mM dithiothreitol (DTT), and 1 mM *N,N,N′,N′*-ethylenediamine tetraacetate (EDTA) were added to each well of a 96-well plastic plate (Corning Inc., Corning, NY, USA). A sample (2.0 μL in MeOH) was added to each well to make the final concentrations from 0 to 4.7–5.6 μM and incubated for 10 min at 37 °C. The reaction was initiated by the addition of pNPP (100 μL of 4.0 mM stock solution) in the citrate buffer, incubated at 37 °C for 30 min, and terminated with the addition of 10 μL of a stop solution (10 M NaOH). The optical density of each well was measured at 405 nm using an MTP-500 microplate reader (Corona Electric Co., Ltd., Ibaraki, Japan). PTP1B inhibitory activity (%) is defined as [1 − (ABS_sample_ − ABS_blank_)/(ABS_control_ − ABS_blank_)] × 100, where ABS_blank_ is the absorbance of wells containing only the buffer and pNPP, ABS_control_ is the absorbance of *p*-nitrophenol liberated by the enzyme in the assay system without a test sample, and ABS_sample_ is that with a test sample. The assays were performed in two duplicate experiments for all test samples. Oleanolic acid (Tokyo Chemical Industry, Tokyo, Japan), a known phosphatase inhibitor [15], was used as a positive control.

### Cytotoxicity assay against HCT-15 and Jurkat cells

HCT-15 and Jurkat cells were obtained from the Center for Biomedical Research, Institute of Development, Aging, and Cancer, Tohoku University (Miyagi, Japan). The cell lines were cultured in RPMI-1640 medium. The medium was supplemented with 10 % fetal bovine serum, 100 U/mL penicillin, and 100 μg/mL streptomycin. Exponentially growing cells, cultured in a humidified chamber at 37 °C containing 5.0 % CO_2_, were used for the experiments.

Cytotoxic activity was evaluated using the colorimetric MTT assay [16]. HCT-15 (1.0 × 10^4^ cells in 100 μL) or Jurkat cells (2.0 × 10^4^ cells in 100 μL) were added to each well of a 96-well plastic plate. A sample (1.0 μL in MeOH) was added to each well to make the final concentrations from 0 to 39–47 μM, and the cells were incubated for 48 h at 37 °C. MTT (10 μL of 5.5 mg/mL stock solution) and a cell lysate solution (90 μL, 40 % *N,N*-dimethylformamide, 20 % sodium dodecyl sulfate, 2.0 % CH_3_COOH and 0.030 % HCl) were added to each well, and the plate was shaken thoroughly by agitation at room temperature for overnight. The optical density of each well was measured at 570 nm using an MTP-500 microplate reader.

### Cytotoxicity assay against Huh-7 cells

Cytotoxic activity against Huh-7 cells was assessed by the MTT assay, a modification of our previously described method [17]. Following the treatment of cells with test samples, 10 μL of MTT (5.0 mg/mL saline) was added to each well, the samples were incubated for 90 min at 37 °C and centrifuged (300*g* for 5 min), and the supernatant was aspirated off. The cells were lysed and solubilized by the addition of 100 μL of 0.040 N HCl in 2-propanol. The absorbance of each well was determined at 590 nm using an Inter-med model NJ-2300 Microplate Reader (Cosmo Bio Co., Ltd., Tokyo, Japan). Survival (%) was calculated relative to the control.

## Results and discussion

Among the ethanol extracts of about 90 marine sponges and ascidians collected in the coral reefs at North Sulawesi, Indonesia, the extract of a marine sponge *Lamellodysidea herbacea* showed potent inhibitory activity (IC_50_ = 0.58 μg/mL) against PTP1B in the screening bioassay. Bioassay-guided isolation by HPLC yielded compound **1** as an inhibitor of PTP1B. The other fractions obtained after separation of **1** did not show an inhibitory activity against PTP1B.

**Fig. 1:**
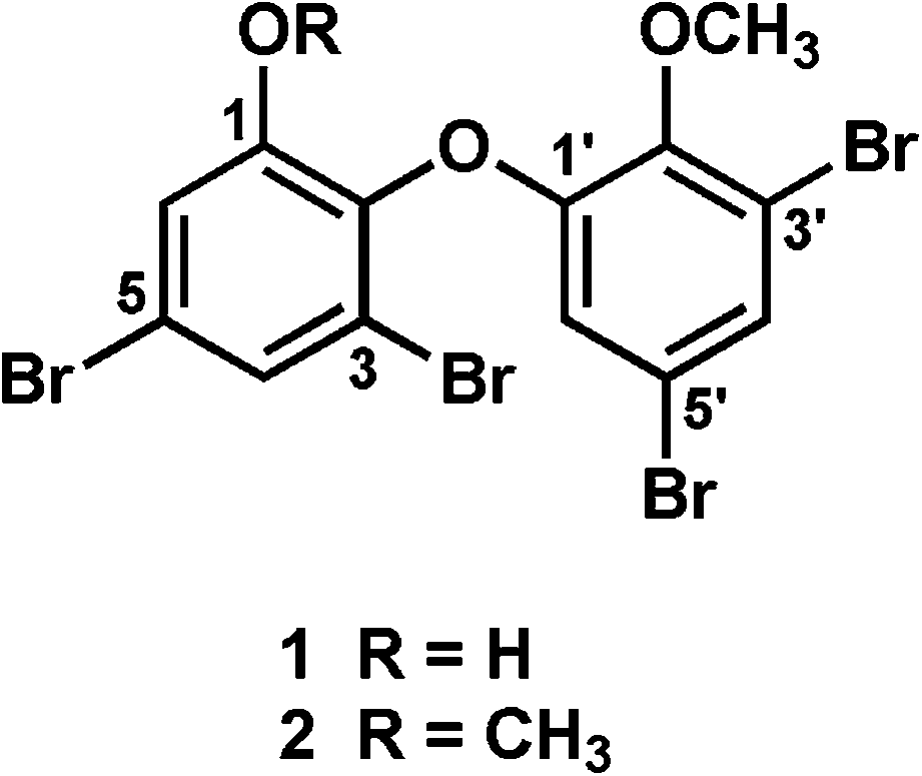
Structures of compounds **1** and **2**

The EI–MS spectrum of **1** showed the presence of four Br atoms, and the molecular formula C_13_H_8_Br_4_O_3_ was deduced from HREI–MS data. The ^13^C NMR spectrum of **1** revealed 13 carbon signals, and the signals due to two sets of meta-coupled aromatic protons (δ 6.80, 7.18, 7.35, and 7.45) and OMe protons (δ 4.03) were detected in the ^1^H NMR spectrum. The positions of an OMe, OH, and four Br atoms were assigned by the analysis of 2D NMR (^1^H–^1^H COSY, HMQC, and HMBC) data for **1** and confirmed by the NOE experiments on the methyl derivative (**2**). The NMR data for **1** were identical with those of the reported values for 2-(3′,5′-dibromo-2′-methoxyphenoxy)-3,5-dibromophenol (Fig. 1) [13].

**Fig. 2:**
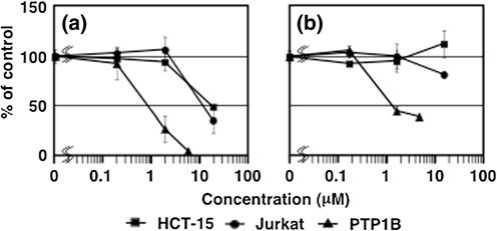
Inhibitory activity of **1** (**a**) and **2** (**b**) against PTP1B and two human cancer (HCT-15 and Jurkat) cells. Data are shown as the mean ± SD (*n* = 4) of two duplicate experiments

Compounds **1** and **2** inhibited the PTP1B activity (Fig. 2) with IC_50_ values of 0.85 and 1.7 μM, respectively, which were almost the same efficacy as that of oleanolic acid (1.1 μM), a positive control (Table 1). Oleanolic acid is a ubiquitous triterpene detected in various plants, most of which are used as crude Asian drugs for the treatments of inflammation, cancers, hepatitis, and diabetes [15, 18, 19], and has recently been reported to have a significant inhibitory activity against PTP1B [20]. Oleanolic acid derivatives were demonstrated to promote cellular insulin signaling by increasing the level of insulin receptor phosphorylation [20]. The highest concentration of compound **2** did not show a dose-dependent effect (Fig. 2). This will be due to a solubility problem of **2** at higher concentration in this bioassay system.

**Table 1:** Inhibitory activity of compounds **1**–**6** against PTP1B and three human cancer cell lines

Compound	IC_50_ (μM)			
	PTP1B	Cytotoxicity		
		Huh-7	HCT-15	Jurkat
**1**	0.85	32	12	9.5
**2**	1.7	48	>46	>46
**3**	0.62	NT	10.3	6.0
**4**	0.68	NT	14.3	9.6
**5**	0.69	NT	7.1	8.1
**6**	0.97	NT	4.3	20
Oleanolic acid	1.1	NT	NT	NT

*NT* not tested

Interestingly, the methylation of a phenol in **1** reduced the cytotoxicity against HCT-15 and Jurkat cells (Fig. 2; Table 1). Compound **1** had a moderate cytotoxicity against HCT-15 and Jurkat cells with IC_50_ values of 12 and 9.5 μM, respectively. On the other hand, **2** did not show an apparent cytotoxicity at 18 μM.

**Scheme 1:**
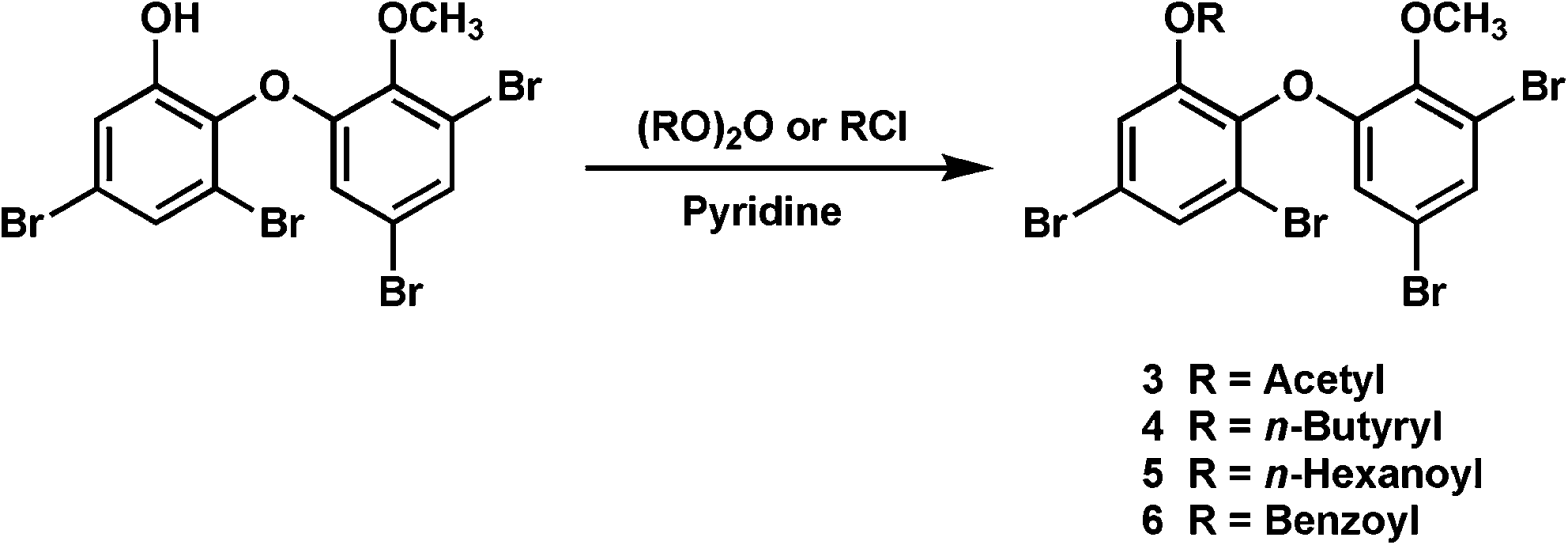
Semisynthetic preparation of **3**–**6**

Therefore, the ester derivatives (**3**–**6**) were prepared from **1** (Scheme 1) and tested for their activity against PTP1B and two cancer cell lines (Table 1). Compound **3**–**6** revealed comparable to stronger inhibitory activity against PTP1B than that of **1**, but cytotoxicity against HCT-15 and Jurkat cells were observed. From these results, **2** is found to be the most interesting compound among these compounds as it possessed potent inhibitory activity against PTP1B and showed much reduced cytotoxicity.

The inhibitory activity of **1** and **2** on cell proliferation of human hepatoma Huh-7 cells was therefore examined. Since PTP1B is located in the insulin-targeted tissues such as liver, muscle, and fat cells, Huh-7 cells are used for cell-based experiments to investigate the mechanism of action of PTP1B inhibitors. Compound **2** showed weaker cytotoxicity (IC_50_ = 48 μM) than **1** (32 μM) (Table 1). Cell-based experiments are now in progress using Huh-7 cells and compound **2**.

Polybrominated diphenyl ethers have been isolated from marine organisms, such as sponges, ascidians, and algae, and are reported to exhibit a variety of biological activities: antibacterial and antifungal activities [21–24], brine shrimp toxicity [23], antimicroalgal activity [25], anti-inflammatory activity [26], maturation of starfish oocytes [27], and inhibitory activities against several enzymes [27–29]. In this study, we demonstrated that a known bromodiphenyl ether (**1**) was a potent inhibitor of PTP1B, an important target enzyme for the treatment of type II diabetes, and that the methoxy derivative (**2**) is more useful than the original phenol and the ester derivatives. Compound **2** will be a new lead compound for PTP1B inhibitors.
